# The impact of social deprivation on the response to a randomised controlled trial of a weight management intervention (BeWEL) for people at increased risk of colorectal cancer

**DOI:** 10.1111/jhn.12524

**Published:** 2017-11-23

**Authors:** A. Fisher, A. M. Craigie, M. Macleod, R. J. C. Steele, A. S. Anderson

**Affiliations:** ^1^ Division of Cancer Research Centre for Research into Cancer Prevention and Screening Ninewells Hospital & Medical School Dundee UK

**Keywords:** deprivation, screening, lifestyle, intervention, colorectal cancer

## Abstract

**Background:**

Although 45% of colorectal cancer (CRC) cases may be avoidable through appropriate lifestyle and weight management, health promotion interventions run the risk of widening health inequalities. The BeWEL randomised controlled trial assessed the impact of a diet and activity programme in overweight adults who were diagnosed with a colorectal adenoma, demonstrating a significantly greater weight loss at 12 months in intervention participants than in controls. The present study aimed to compare BeWEL intervention outcomes by participant deprivation status.

**Methods:**

The intervention group of the BeWEL trial (*n* = 163) was classified by the Scottish Index of Multiple Deprivation (SIMD) quintiles into ‘more deprived’ (SIMD 1–2, *n* = 58) and ‘less deprived’ (SIMD 3–5, *n* = 105). Socio‐economic and lifestyle variables were compared at baseline to identify potential challenges to intervention adherence in the more deprived. Between group differences at 12 months in primary outcome (change in body weight) and secondary outcomes (cardiovascular risk factors, diet, physical activity, knowledge of CRC risk and psychosocial variables) were assessed by deprivation status.

**Results:**

At baseline, education (*P* = 0.001), income (*P* < 0.001), spending on physical activity (*P* = 0.003) and success at previous weight loss attempts (*P* = 0.007) were significantly lower in the most deprived. At 12 months, no between group differences by deprivation status were detected for changes in primary and main secondary outcomes.

**Conclusions:**

Despite potential barriers faced by the more deprived participants, primary and most secondary outcomes were comparable between groups, indicating that this intervention is unlikely to worsen health inequalities and is equally effective across socio‐economic groups.

## Background

Colorectal cancer (CRC) is the third commonest cancer in Scotland 1 and it is estimated that 45% of cases could be avoidable by appropriate lifestyle changes 2. In Scotland, CRC risk is associated with increasing deprivation in men 3, which may be partly attributable to a higher BMI, as well as smoking 4. A low socio‐economic status has also been linked with a reduced consumption of fruit, vegetables, wholemeal bread and fibre and an increased consumption of fat, meat, processed meat and sugar 5, 6, 7, 8, 9, 10, 11, 12. However, those individuals from more deprived backgrounds may also face a wide range of barriers to a healthy lifestyle, such as food prices 13, 14, 15, 16, 17, a lack of local facilities 18, pre‐existing health problems 19 , lower education 11 and lower self‐efficacy 20, 21.

The BeWEL trial 22 assessed the impact of a lifestyle (body weight, diet and physical activity) intervention following removal of a colorectal adenoma amongst people participating in the Scottish Bowel Screening programme (aged 50–74 years) who had a body mass index (BMI) > 25 kg/m^2^. The 12‐month intervention involved three face‐to‐face visits with a lifestyle counsellor and nine telephone consultations at monthly intervals. The primary outcome of the trial was change in body weight. Secondary outcomes included markers of cardiovascular and diabetes risk, diet and physical activity, knowledge of colorectal cancer risk factors, and psychosocial factors including quality of life and self‐efficacy. Compared to the control group, the intervention group succeeded in losing significantly more weight and making lifestyle changes consistent with a reduction in risk of adenoma recurrence and the development of CRC.

Despite the positive changes found following the BeWEL intervention, there remains a concern that such lifestyle interventions run the risk of widening health inequalities if they are more effective in higher socio‐economic groups. Those from more deprived backgrounds may be less successful in behaviour change, harder to recruit to interventions 23, 24, 25 and have higher dropout rates 26. The present study therefore aimed to identify potential barriers to successful lifestyle changes experienced by the more deprived at baseline and to compare the outcomes of the BeWEL intervention by participant deprivation status.

## Materials and methods

### Recruitment

Recruitment to the BeWEL trial took place between November 2010 and May 2012, across four National Health Service (NHS) health boards (Tayside, Forth Valley, Ayrshire and Arran, and Greater Glasgow and Clyde). Scottish Bowel Screening participants, aged 50–75 years, who had undergone polypectomy for adenoma, were approached by letter. Eligibility criteria were BMI > 25 kg/m^2^, the ability to be physically active and the absence of insulin dependent diabetes, pregnancy or any cancer diagnosis. Of the 997 people approached, 329 were recruited, with 163 being randomised to intervention and 166 to control. Most participants were male (74%), reflecting the higher rate of adenoma detection in men 27, and a significant proportion (35%) came from the two most deprived Scottish Index of Multiple Deprivation (SIMD) quintiles (SIMD 1–2). Full details of the recruitment process are available elsewhere 28.

### Randomisation

Participants were randomised (1:1) to parallel groups, using a permuted‐block technique, to either the control or intervention group.

### Intervention

The intervention group received three 1:1 lifestyle counsellor coaching sessions, monthly telephone calls, a personalised energy prescription [25.104 MJ (600 kcal) below that required for weight maintenance] and a weight loss booklet: the British Heart Foundation publication ‘So you want to lose weight for good’ 29. Motivational interviewing techniques were used to explore self‐assessed confidence, ambivalence and personal values regarding weight and participants were encouraged to set goals and self‐monitor their progress. They were also provided with a pedometer and body weight scales. Tools such as skipping ropes and exercise videos were made available for loan. The control group received the weight loss booklet only. The intervention has been described in detail elsewhere 30.

The primary outcome was weight change, with intervention participants being set a goal weight loss of 7% of their starting bodyweight. Secondary outcomes were waist circumference, blood pressure, fasting cardiovascular and glucose metabolism biomarkers, physical activity, diet and alcohol consumption changes, and self‐reported psychosocial variables at 12 months. The full protocol for the BeWEL trial is available elsewhere 31.

### Baseline and follow‐up measures

Sociodemographic data, including age, sex, marital status, education and employment, as well as spending on groceries, physical activity and previous attempts at weight loss, were recorded at baseline. The postcode of each participant was used to calculate the SIMD quintile in which they lived. The measure not only represents geographical area *per se*, but also is a composite, categorical system of identifying deprivation based on area of residence, which takes account of housing, crime, access to services, education, health, income and employment 32.

At baseline, 3 months and 12 months, body measurements (height, weight, waist circumference), cardiovascular and glucose metabolism markers and physical activity were measured. Self‐reported diet, knowledge of CRC risk factors and psychosocial variables were assessed using a questionnaire. Cardiovascular and glucose metabolism markers included systolic and diastolic blood pressure, total cholesterol, high‐density lipoprotein (HDL) cholesterol, low‐density lipoprotein cholesterol, triglycerides, glucose, insulin, HOMA (homeostasis model assessment) and HbA1c. Blood samples were taken after fasting for 12 h.

Physical activity was measured by daily step count, and time spent in sedentary [<3 metabolic equivalents (MET)], moderate (3 to <6 MET) and vigorous (≥6 MET) activity, using a SenseWear monitor (BodyMedia, Pittsburgh, PA, USA). The DINE (Dietary Instrument for Nutrition Education) questionnaire was used to calculate scores for fat, unsaturated fat and fibre consumption 33. Fat scores could range from 7 to >77 and were based on the frequency of consumption of foods that contribute substantially to fat intake (dairy, meat, processed meat, fried fish, fried foods, sweet and savoury snacks, and fat spreads). Scores below 30 were equivalent to a fat intake of ≤83g per day (<35% of total energy intake for an average woman). Unsaturated fat scores could range from 3 to 12 and were based on the type of fats used. A score of up to 5 was considered ‘low’ and a score of 10 or more was considered ‘high’. Fibre scores could range from 3 to 88 and were based on the frequency of intake of bread, rice, potatoes, pasta and other starchy foods, and fruit and vegetables (including beans and lentils). A score of less than 30 (low) was equivalent to a fibre intake of 20g per day or less, whereas a score of more than 40 (high) was equivalent to an intake of more than 30g per day. The two‐item questionnaire of Cappuccio *et al*. 34 was modified and used to estimate daily fruit and vegetable portions. Sugary drink intake was measured using nine frequency categories and questions from the AUDIT (Alcohol Use Disorders Inventory Test) questionnaire were used to monitor alcohol consumption 35.

Knowledge of CRC risk factors was explored using the question ‘What do you personally think are the main factors that might increase or decrease a person's chance of developing colorectal cancer?’. Answers were coded and scored with body fatness, alcohol, red meat, processed meat, physical activity/exercise and fibre all receiving a score of +1. Fruits and vegetables and/or cereals/whole grains/pulses and sedentary activity scored +0.5. The maximum possible score was +6 36.

### Subgroup analysis

A subgroup analysis was performed on the intervention cohort of the trial. Intervention participants were grouped into ‘more deprived’ and ‘less deprived’ based on their SIMD quintile. Those who lived in SIMD quintiles 1–2 were classed as ‘more deprived’ and those from SIMD quintiles 3–5 as ‘less deprived’. To identify potential barriers to lifestyle change, baseline demographics, spending on groceries and physical activity, and previous successful weight loss attempts were compared between deprivation groups. Changes in lifestyle, body measurements, cardiovascular and glucose metabolism markers, knowledge of CRC risk factors, household weekly spending on groceries, and physical activity throughout the intervention were also compared between groups.

Where no between group differences were found, variables were also compared from 12 months to baseline within each group, aiming to identify changes within groups.

All analyses were performed using spss, version 22.0 (IBM Corp., Armonk, NY, USA). Kolmogorov–Smirnov tests were used to evaluate whether each variable was normally distributed. Normally distributed continuous variables are reported as the mean (SD) and independent *t*‐tests were used to compare groups. For nonparametric data, Mann–Whitney tests were used to compare groups. Categorical data were reported as number (percentage) and chi‐squared tests and odds ratios were used to test for between group differences in proportions, as well as the magnitude of any differences, respectively. For within group differences in repeated measures, paired *t*‐tests (for normally distributed data) or Wilcoxon signed rank tests were used.

### Ethical approval

Ethical approval for the present study was provided by the Tayside Committee on Medical Research Ethics B on 16 July 2010 (REC Reference No. 10/S1402/34).

## Results

In this cohort (*n* = 163), most participants were male, married or co‐habiting and not in employment (Table 1). One‐third (36%) of participants came from the two more deprived SIMD quintiles (SIMD 1–2). The proportion of participants whose highest level of qualification was from primary or secondary school was significantly higher in the more deprived category than the less deprived (56.9% versus 29.5%, *P* = 0.001). In addition, the proportion of participants with a household income of <£25 000 per year was higher in SIMD 1–2 than SIMD 3–5 (34.5% versus 21.2%, *P* < 0.001). A greater proportion of SIMD 1–2 (17.2%) than SIMD 3–5 (4.8%) were smokers at baseline [*P* = 0.008, odds ratio = 4.17, 95% confidence interval (CI) = 1.35–12.86].

**Table 1:** Baseline demographic characteristics by Scottish Index of Multiple Deprivation (SIMD) deprivation category

Baseline characteristics	SIMD 1–2 (more deprived) (*n* = 58)	SIMD 3–5 (less deprived) (*n* = 105)	All (*n* = 163)
Age (years)	64	63	63
Median (LQ, UQ)	(59,71)	(59,68)	(59, 69)
Range	50‐75	50‐75	50–75
Sex			
Male, *n* (%)	45 (77.6)	75 (71.4)	120 (73.6)
Female, *n* (%)	13 (22.4)	30 (28.6)	43 (26.4)
Marital status			
Married or cohabiting, *n* (%)	42 (72.4)	88 (83.8)	130 (79.8)
Single, divorced, widowed or separated, *n* (%)	16 (27.6)	17 (16.2)	33 (20.2)
Employment status			
Employed (full or part time), *n* (%)	20 (34.5)	43 (41.0)	63 (38.7)
Unemployed, *n* (%)	1 (1.7)	1 (1)	2 (1.2)
Retired, student or other, *n* (%)	37 (63.8)	61 (58.1)	98 (60.1)
Income			
Household income >£25 000 year^−1^, *n* (%)	36 (65.5)	87 (88.8)	123 (80.4)
Highest educational attainment			
Primary or secondary school, *n* (%)	33 (56.9)	31 (29.5)	64 (39.3)

LQ, lower quintile; UQ, upper quintile. Quintile 1 = most deprived; quintile 5 = least deprived.

Household weekly spending on physical activity was lower at baseline in SIMD 1–2 (median: 0; lower quintile: 0, upper quintile: 5; range: 0–60) than SIMD 3–5 (median: 3; lower quintile: 0, upper quintile: 20; range: 25–200), *P* = 0.003. Fewer participants from SIMD 1–2 increased this spending by 12 months (7.8% versus 20.6%), *P* = 0.045. Median household weekly spend on groceries (excluding alcoholic drinks) did not vary significantly between groups (median: 70; lower quintile: 50, upper quintile: 100; range: 1–200). Overall, 56.8% had increased their spending on groceries by the end of the intervention and this did not vary by deprivation group.

### Primary outcome

Weight change (primary outcome), BMI and waist circumference of participants at baseline did not differ significantly by deprivation category, and almost half (47.9%) were obese at baseline. The proportion who had experienced previous weight loss success was higher in SIMD 3–5 (60%) than SIMD 1–2 (37.9%) (*P* = 0.007). Despite this, no significant difference was detected in weight, BMI or waist circumference changes between deprivation groups at 12 months (Table 2). In both groups, weight, BMI and waist circumference were significantly lower at 12 months than baseline (*P* < 0.001). One‐fifth (22%) met the 7% body weight loss target and 36% lost 5% body weight. Trial retention (at 90.8%) also did not vary significantly by deprivation status.

**Table 2:** Changes in anthropometric measures from baseline to 12 months by Scottish Index of Multiple Deprivation (SIMD) deprivation category

Baseline and follow‐up measures	SIMD 1–2 (more deprived)			SIMD 3–5 (less deprived)			Between group difference *P* value
	*n*	Median (LQ, UQ) range	Difference to baseline	*n*	Median (LQ, UQ) range	Difference to baseline	
Weight (kg)							
Baseline	58	88.0 (80.9, 101.4) 63.0‐133.4	–2.80 (–5.50, –0.90)	105	86.5 (80.0, 96.9) 62.3–141.1	–2.80 (–6.20, –2.80)	0.83
12 months	51	84.6 (76.7, 99.1) 61.3–131.9		97	84.0 (75.2, 94.5) 61.0–136.5		
BMI (kg/m^2^)							
Baseline	58	30.3 (27.9, 35.1) 25.5–46.8	–0.89 (–1.98, –0.30)	105	29.8 (27.9, 32.4) 25.0–47.4	–0.91 (–2.10, –0.10)	0.90
12 months	51	29.1 (26.9, 33.1) 24.0–45.1		97	28.3 (26.4, 30.7) 24.4–45.0		
Waist circumference (cm)							
Baseline	58	102.1 (95.7, 115.0) 86.3–139.0	–3.80 (–6.00, –1.20)	105	102.7 (97.5, 109.3) 82.0–129.6	–4.25 (–8.43, –2.00)	0.16
12 months	51	98.1 (93.9, 111.2) 75.2–133.0		94	96.2 (90.9, 104.8) 76.5–128.0		

BMI, body mass index; LQ, lower quintile; UQ, upper quintile. Quintile 1 = most deprived; quintile 5 = least deprived.

### Secondary outcomes

Many (20.3%) participants had type 2 diabetes and almost half (48%) were hypertensive at baseline, with no difference by deprivation category for either variable. The only difference between categories for baseline cardiovascular and glucose metabolism markers was HDL cholesterol, which was significantly lower in SIMD 1–2 than 3–5 (mean 1.23, 95% CI = 1.05–1.40 versus mean 1.34, 95% CI = 1.21–1.57 respectively, *P* = 0.011). Changes in cardiovascular and glucose metabolism markers at 12 months did not vary by SIMD group.

There were no differences between deprivation groups in diet and physical activity variables at baseline, or in their change over 12 months, with the exception of unsaturated fat consumption, which was lower in SIMD 1–2 at baseline (*P* = 0.037) (Table 3).

**Table 3:** Changes in (a) daily average physical activity (b) dietary intake by Scottish Index of Multiple deprivation (SIMD) deprivation category

Baseline and follow up measures	SIMD 1–2 (more deprived)			SIMD 3–5 (less deprived)			Between group differences *P* value
	*n*	Median (LQ, UQ) range	Difference to baseline, median (LQ, UQ) *P* value	*n*	Median (LQ, UQ) range	Difference to baseline, median (LQ, UQ) *P* value	
*Daily average physical activity*							
Time spent in sedentary activity (min/day)^a^							
Baseline	53	1326 (1270, 1372) 1008–1433	–6 (–70, 33) 0.20	100	1325 (1228, 1371) 634–1420	–10 (–58, 40) 0.53	0.44
12 months	49	1326 (1246, 1374) 266–1434		88	1319 (1261, 1373) 456–1420		
Time spent in moderate activity (min/day)^b^							
Baseline	53	62 (40, 119) 0–238	2 (–23, 29) 0.55	100	63 (39, 111) 1–356	7.5 (–19, 37) 0.17	0.68
12 months	49	63 (40, 120) 2–324		88	79 (41, 114) 1–294		
Step count							
Baseline	55	7110 (4718, 9940) 7110–16 031	–215 (–1187, 1504) 0.71	104	8202 (5921, 10 758) 1421–25 178	63 (–983,1380) 0.42	0.84
12 months	49	7067 (5070, 10 664) 328–22 468		91	8415 (6380, 11 955) 911–25 513		
*Dietary intake*							
Fruit and vegetable (portions/day)							
Baseline	58	3 (2, 5) 0–10	0 (–1, 2) 0.52	105	4 (3, 5) 0–12	1 (–1, 2) **<0.001**	0.08
12 months	51	4 (2, 5) 0–11		97	5 (3, 6) 0–11		
Unsaturated fat score							
Baseline	58	9 (8, 10) 3–11.0	0 (0, 2) **0.003**	103	9 (8, 10) 3–11	0 (–1, 1) **0.049**	0.21
12 months	50	9 (8, 10) 6–11		97	9 (8, 10) 5–11		
Fibre consumptions score							
Baseline	58	32 (26, 38) 10–56	–3 (–7, 4) 0.27	105	33 (26, 41) 12.0–63.0	0.0 (–5, 6) 0.71	0.23
12 months	51	30 (23, 37) 14–50		97	33 (26, 40) 11–78		
Total fat consumptions score, mean (SD)							
Baseline	58	32.2 (12.1)	–8.6 (12.9) **<0.001**	105	29.4 (9.6)	–6.6 (8.3) **<0.001**	0.27
12 months	51	24.4 (7.8)		95	23.2 (7.1)		

LQ, lower quintile; UQ, upper quintile. Quintile 1 = most deprived; quintile 5 = least deprived.

a Sedentary activity: <3 metabolic equivalents (MET).

b Moderate activity: 3–5 MET.

At baseline, a greater proportion of those in SIMD 1–2 consumed sugary drinks than in SIMD 3–5 (25.9% versus 12.4%, *P* = 0.029). Most of the cohort (78.3%) reduced their intake of sugary drinks by 12 months and this did not vary by SIMD group. Reduction in alcohol consumption frequency and weekend/weekday amount was also comparable by SIMD group. By 12 months, 29.7% of alcohol consumers reported reducing the frequency of their alcohol consumption, 29.7% reduced the amount they drank on weekdays and 21% reduced their intake at weekends.

At baseline, the most well‐known CRC risk factor was physical activity, correctly identified by 49.7% of participants, followed by alcohol (44.8%), lack of foods containing fibre (35%), body fatness (15.3%), red meat (12.9%) and processed meat (1.2%). The proportion of participants correctly identifying each risk factor did not vary by deprivation group. Overall, 9.2% could not identify any risk factors at baseline, regardless of SIMD group. The median knowledge score was 1.5 (lower quintile: 1, upper quintile: 2, range: 0–5) at baseline and no significant difference was detected by deprivation category. Change in knowledge score was also comparable between groups.

**Table 4:** Acquired knowledge of colorectal cancer risk factors at 12 months by Scottish Index of Multiple deprivation (SIMD) deprivation category

Risk factor	*n*	Acquired knowledge at 12 months *n* (%)	Odds ratio (95% CI) *P* value
Foods containing fibre			
SIMD 1–2	51	9 (17.6)	0.94 (0.39‐2.28) 0.89
SIMD 3–5	97	18 (18.6)	
Physical activity			
SIMD 1–2	51	10 (19.6)	1.24 (0.52‐2.96) 0.64
SIMD 3–5	97	16 (16.5)	
Alcohol			
SIMD 1–2	51	10 (19.6)	1.24 (0.52‐2.96) 0.64
SIMD 3–5	97	16 (16.5)	
Body fatness			
SIMD 1‐2	51	9 (17.6)	1.68 (0.65‐4.35) 0.28
SIMD 3‐5	97	11 (11.3)	
Red meat			
SIMD 1‐2	51	2 (3.9)	0.24 (0.05‐1.11) 0.05
SIMD 3‐5	97	14 (14.4)	
Processed meat			
SIMD 1‐2	51	0 (0.0)	0.55^a^
SIMD 3‐5	97	3 (3.1)	

CI, confidence interval. 1 = most deprived, 5 = least deprived.

a *P* value only for processed meat.

After 12 months, a new awareness of the link between dietary fibre and CRC was seen in 18.2% of participants, physical activity in 17.6% of participants, alcohol in 17.6%, body fatness in 13.5% and processed meat in 2% (Table 4). The proportion of participants who acquired knowledge of these risk factor did not vary by deprivation status. There were no between group differences detected in acquired knowledge of processed meat as a risk factor and knowledge in both groups remained low at 12 months.

## Discussion

The BeWEL study had high recruitment and retention rates from deprived groups, with 35% coming from people living in SIMD 1–2 areas. This is noteworthy because low income groups can often be more difficult to recruit to lifestyle interventions 23, 24, 25 and may have higher dropout rates 26. Although this demographic distribution is a strength of the overall study, the present analyses focuses on the intervention arm only, which is a subgroup study and therefore only indicative outcomes can be identified.

The results of the trial were comparable between groups for all primary and main secondary outcomes, indicating that the BeWEL intervention was equally effective across the deprivation gradient. Both groups showed comparable improvement in anthropometric measures, lifestyle variables and self‐efficacy. This is supported by a previous meta‐analysis suggesting that lifestyle interventions aimed at managing obesity do not worsen healthcare inequalities 37.

Differences were identified between groups at baseline that could act as barriers to successful lifestyle change in the more deprived group. The more deprived were less likely to have achieved weight loss prior to the study and had lower income and educational levels at baseline, all of which have been previously described as barriers to lifestyle change 13, 14, 15, 16, 17, 18. Despite this, the more deprived managed to perform comparably with the rest of the cohort. This may be attributable, in part, to the study design, which offered free scales to aid self‐ monitoring and exercise equipment, such as skipping ropes and exercise videos to participants. Emphasis was also put on walking as an inexpensive way to increase physical activity and decrease sedentary time. This finding supports the evidence that individual weight management interventions, such as BeWEL, do not worsen health care inequalities in participants 37.

## Transparency declaration

The lead author affirms that this manuscript is an honest, accurate and transparent account of the study being reported. The reporting of this work is compliant with CONSORT guidelines. The lead author affirms that no important aspects of the study have been omitted and that any discrepancies from the study as planned (Current Controlled Trials ISRCTN53033856) have been explained.

**Conflict of interests, source of funding and authorship**

The authors declare that they have no conflicts of interests.

This study was funded by the MRC National Prevention Research Initiative (www.npri.org.uk), grant award No G0802030. National Prevention Research Initiative is a national research initiative administered by the Medical Research Council made up of the following funding partners: Alzheimer's Research Trust; Alzheimer's Society; Biotechnology and Biological Sciences Research Council; Cancer Research UK; Chief Scientist Office, Scottish Government Health Directorate; Department of Health; Diabetes UK; Economic and Social Research Council; Engineering and Physical Sciences Research Council; Health and Social Care Research and Development Office for Northern Ireland; Medical Research Council; Welsh Assembly Government; and World Cancer Research Fund. Further financial support was provided by NHS Research Scotland to carry out this work. The Health Services Research Unit, University of Aberdeen, is core funded by the Chief Scientist Office of the Scottish Government Health Directorates.

All authors contributed to the preparation of the manuscript. ASA and RJCS (guarantors) had the original idea for the trial and carried out the trial design with AMC, formed the investigator group that obtained the funding, as well as oversaw the study implementation and data collection. AF carried out the analysis reported in this paper, under the supervision of ASA, AMC, MM and RJCS, and also prepared the initial draft of the manuscript. All authors critically reviewed the manuscript and approved the final version submitted for publication.
